# Awareness of and the relationship between noise-induced hearing loss and the use of personal listening devices in Jazan region, Saudi Arabia

**DOI:** 10.3389/fpubh.2025.1505442

**Published:** 2025-02-21

**Authors:** Mohammad M. Mokhatrish, Ramzi M. Dighriri, Abdulrahman A. Otaif, Abdulelah A. Otaif, Riyadh A. Jahlan, Abdulrahman A. Daghreeri, Hussam T. Hakami, Ameer M. Mobarki, Bandar F. Daghriri, Fawaz M. Hakami, Osama M. Dighriri

**Affiliations:** ^1^Department of Otolaryngology-Head and Neck Surgery, College of Medicine, Prince Sattam Bin Abdulaziz University, Alkharj, Saudi Arabia; ^2^Department of Otolaryngology-Head & Neck Surgery, Armed Forces Hospital, Jazan, Saudi Arabia; ^3^Faculty of Medicine, Jazan University, Jazan, Saudi Arabia; ^4^Faculty of Nursing, Jazan University, Jazan, Saudi Arabia

**Keywords:** awareness, knowledge, personal listening devices, NIHL, Jazan, Saudi Arabia

## Abstract

**Objectives:**

Noise-induced hearing loss (NIHL) is a significant global health issue, exacerbated by the increasing use of personal listening devices (PLDs). This study aims to assess the awareness of NIHL and its association with PLD use among residents in the Jazan region of Saudi Arabia.

**Materials and methods:**

A descriptive, cross-sectional study was conducted among Saudi adults in Jazan. A structured questionnaire was used to gather data on demographics, health-related characteristics, PLD usage patterns, and awareness of NIHL.

**Results:**

The study included 428 participants, with 53.3% males and 45.6% aged 18–25. Most participants used PLDs frequently, with 43.0% listening for an hour or less daily. Awareness of NIHL was moderate, with 81.1% recognizing the harmful effects of loud noise on hearing. However, misconceptions about early signs and prevention of hearing loss (HL) were prevalent. The mean HL score was 8.9 (SD: 2.8) out of 20; which reflects low level of HL among the study participants. Males were 1.6 folds more likely to have higher level of HL compared to females (*p* = 0.015). Participants who prefer high volume level (above 70 dB) were more than 2-folds likely to have higher level of HL compared to others (*p* < 0.01).

**Conclusion:**

The study highlights moderate level of awareness of NIHL and the risks of PLDs in Jazan region of Saudi Arabia. However, implementing preventive measures, especially among younger demographics, remains a challenge. The findings suggest the need for targeted public health interventions and technology to strengthen hearing conservation strategies.

## Introduction

Hearing loss (HL) has become one of the most common disabilities globally, affecting 6.1% of the global population, and impacting the ability to differentiate sounds and engage in conversations. It also adversely affects emotional health and social well-being (1, 2). Causes of HL vary by age: in children, it often stems from infection or genetic factors, while in adults, it is frequently due to aging and prolonged noise exposure (3, 4). Noise-induced hearing loss (NIHL) results from damage to auditory structures due to exposure to loud sounds from occupational, environmental, or recreational sources (5–7). The increasing use of smartphones and personal listening devices like earphones and headphones has raised concerns about NIHL (5, 8, 9). In the current period, noises from recreation are more common, even though noise from work might often be more dangerous (7). Exposure to noise at 85 dB for at least 8 h per day may result in permanent HL (10). Therefore, Misuse of these devices can lead to difficulty in understanding speech, tinnitus, unsteadiness, and reduced hearing capability (11).

Studies across different regions of Saudi Arabia indicate varying levels of awareness and prevalence of NIHL. In Makkah, 22% of subjects had mild-to-severe hearing impairment, with most preferring lower volume levels as a preventive measure (12). In Hail, those exposed to workplace noise had more significant hearing impairments due to low awareness levels (13). In Jouf, a significant portion lacked knowledge about NIHL, with 40.4% reporting that noise from personal listening devices affected those around them (14). A nationwide study revealed inadequate knowledge about NIHL, with 25% of subjects reporting mild-to-severe hearing problems (15). Another study among medical students showed poor general knowledge about NIHL, with only 18.3% aware that it is not a type of conductive HL (16). In the Eastern Province, over half the participants were unaware of the health risks associated with personal audio devices (17).

Regionally, a study in Jordan found that only 9.8% of university students used earplugs despite recognizing HL as a serious issue (18). Internationally, a study in Malaysia linked the use of personal listening devices among high school students to a risk of HL, particularly among males who listened at higher volumes (19). In Singapore, 16% of young people used portable music players at potentially harmful volume levels (20).

Nowadays the prevalence of NIHL is increasing due to the use of smartphones and personal listening devices. Therefore, our rationale is to evaluate the awareness level about the relationship between noise-induced HL and the use of personal listening devices in Jazan Region, Saudi Arabia. Specifically, this research aimed to answer the following research question: What is the level of awareness regarding the impact of personal listening devices (PLDs) on NIHL, and what is the influence of demographic characteristics on their awareness level?. In addition, to the best of our knowledge, no studies have been conducted on a similar topic in Jazan region. The geographical, cultural, and socioeconomic characteristics of the Jazan region are distinct from those of Jouf, Hail, and Makkah, where previous research has been conducted. PLDs utilization pattern and their associated NIHL awareness and prevalence may be influenced by the following characteristics: a more traditional lifestyle, a lower income level, and a lack of prominent public health initiatives. Thus, it is crucial to conduct this study for the development of effective preventive strategies and interventions in our region. The findings of this study offer insights specific to the region’s cultural and behavioral patterns in Jazan region, Saudi Arabia. Moreover, it provides evidence to inform targeted interventions and policies to reduce the burden of NIHL in this population.

## Materials and methods

### Study design and study population

This is a descriptive, cross-sectional study aimed to evaluate the level of awareness regarding NIHL and the utilization of PLDs within the Jazan Region’s populace. Jazan City, positioned along the Red Sea coast, was identified as one of Saudi Arabia’s rapidly expanding urban centers. As per the 2017 Census, the city boasted a population of 1,567,547 inhabitants and covered an area spanning 11,671 km^2^.

The study targeted Saudi adult residents of the Jazan region aged 18 years and above, Arabic speaking, and consenting to participate. Individuals declining participation, non-Arabic speaking, non-residents of Jazan, and those younger than 18 years old were excluded.

### Data collection tool

A previously developed questionnaire was used in this study after obtaining consent from the corresponding author for a study conducted in Hail, Saudi Arabia (13). Multiple otolaryngologists developed and validated the original questionnaire to evaluate community awareness of NIHL from PLDs. In order to validate the language, two independent translators translated the questionnaire from English to Arabic and then back to English. First, pilot research was carried out to statistically assess the survey’s reliability using Cronbach alpha, which deemed reliable with *α* >0.70 (13). The questionnaire included a total of 37 items distributed into six categories. The initial section consisted of six items to collect personal data (Demographics - gender, age, nationality, marital status, location of residence, level of education, and employment). The second section contained five items about medical history (Health-related characteristics such as comorbidities, smoking history, and NIHL in the family). The third section comprised five items about the utilization of PLDs [Individual listening device usage patterns such as the type of headphones, frequency and duration of use, and volume settings (volume level was determined based on individuals’ devices numerical scale; as each participant utilized a PLD that permitted them to modify and display the volume as a number between 0 and 100 when we state that the volume level was determined based on the numerical scale of their devices. The volume level was assessed by recording the specific number that each participant reported out of 100, rather than measuring the sound in decibels)]. Similarly, the fourth section had five items and was designed to evaluate the symptoms of hearing impairment. In order to quantify the level of HL, we used 4-point Likert scale (ranged from 1 for “never” to 4 for “always”) for the five items that evaluated the symptoms of hearing impairment. The higher the score, the higher the level of HL. The fifth section contained a total of 11 items that were used to assess the knowledge and beliefs regarding NIHL. Lastly, the last section consisted of five items about the protective measures to prevent NIHL.

### Data collection process

This study employed convenience (non-random) sampling technique. The use of convenience sampling technique facilitates participants recruitment across wide geographical area within considerable time and effort. The data was collected through a self-administered online questionnaire that was disseminated using social media platforms (WhatsApp, Telegram, X, and others). After data collection, the data was verified manually, and then coding was carried out within an Excel sheet. The study aim and objectives were highlighted in the invitation letter of the questionnaire. Participants who meet the inclusion criteria were asked to participate in the study.

### Statistical analysis

This study employed descriptive statistics to collect data on personal listening device usage habits, health-related characteristics, and demographics. Categorical variables were represented using frequency distributions and percentages. Chi-square tests were used to examined the difference in participants behaviors and lifestyle in terms of their demographic characteristics. Binary logistic regression analysis was used to identify predictors of higher level of HL. The cut-off point used to identify the dummy dependent variable for the regression analysis was 8.9 (which is the mean HL score for the study participants). Therefore, participants with a total HL score of 9 or higher were considered as having higher likelihood of experiencing more severe HL indicated by having higher HL score.

The use of mean score as the cut-off point in the regression model provides objective and central value that provide balanced comparison across the study sample.

### Sample size

The sample size was determined using Raosoft software accessed at http://www.raosoft.com/samplesize.html. Based on data from the General Authority for Statistics approximating the region’s population at 1,404,997 individuals, a sample size was calculated with a 95% confidence level, assuming a 50% response rate, and a margin of error of ±5%. Initially set at 385, the minimum required sample size was adjusted to 424 to factor in a 10% allowance for non-responses.

## Results

Table 1 displays the demographic characteristics of participants in the Jazan region of Saudi Arabia. Males comprised 53.3% of the participants, 45.6% were aged 18 to 25, and 97.7% were Saudi citizens. More than half of the participants (55.1%) were single, and the sample population distribution was evenly split between cities (50.2%) and villages (49.8%). Of the participants, 70.1% obtained a university degree, and 62.9% did not work in the health field.

**Table 1:** Demographics of research participants.

Demographic characteristics		*N*	%
Gender	Male	228	53.3%
	Female	200	46.7%
Age	18–25	195	45.6%
	26–35	114	26.6%
	36–50	93	21.7%
	Over 50	26	6.1%
Nationality	Saudi	418	97.7%
	Non-Saudi	10	2.3%
Marital status	Single	236	55.1%
	Married	192	44.9%
Residence	City	215	50.2%
	Village	213	49.8%
Education level	Middle school or below	22	5.1%
	Secondary	77	18.0%
	University	300	70.1%
	Postgraduate	29	6.8%
Employment	Health practitioner	159	37.1%
	Outside the health field	269	62.9%

Table 2 shows the health-related features of the participants and their noise exposure. Most individuals (72.7%) did not smoke, with 19.2% currently smoking and 8.2% quitting smoking. Most participants (87.1%) had no long-term medical issues, including heart disease, diabetes, or hypertension. Among the participants, 85.7% reported no HL, 9.3% had mild impairment, 4.4% had moderate impairment, and 0.5% had severe impairment. More than half (55.1%) of the participants did not know someone close “family member or friend” who has HL. Thirty-seven percent of the interviewees reported working in a noisy environment or loud noise.

**Table 2:** Participants’ health characteristics.

Health characteristics		*N*	%
Do you smoke?	No	311	72.7%
	Yes	82	19.2%
	Quit smoking	35	8.2%
Do you have one of these diseases?	Diabetes	23	5.4%
	Hypertension	24	5.6%
	Heart disease	8	1.9%
	None	373	87.1%
Have you been diagnosed with hearing loss?	Mild impairment	40	9.3%
	Moderate impairment	19	4.4%
	Severe impairment	2	0.5%
	No impairment	367	85.7%
Do you know anyone close “family member or friend” to you who has a hearing loss?	No	236	55.1%
	Yes	192	44.9%
Are you exposed to noise or loud noise in your field of work?	No	271	63.3%
	Yes	157	36.7%

Table 3 displays the preferences and usage patterns of participants’ personal listening devices. The participants’ top choices were large external speakers (8.9%), automotive speakers (17.1%), headphones (19.4%), and earbuds (54.7%). Around43.0% of the participants used these devices one to five times each week, with 24.8% using them six to nine times, and 21.5% using them more than ten times. Only 10.5% of the polled had never used their own listening equipment. Forty three percent of the participants wore their headphones for an hour or less every day, followed by 31.1% for 1–2 h, 16.4% for 3–5 h, and 9.6% for more than 5 h. The majority of participants (57.7%) claimed that the sounds emitted by their devices did not affect anyone close to them, whereas 31.8% stated that this was occasionally the case. Most participants preferred lower volume levels, with 27.8% using levels ranging from 0 to 49, 21.3% from 50 to 59, and 17.8% from 60 to 69. In contrast, 9.3% of the participants used volume levels ranging from 80 to 89, 8.2% from 90 to 100, and 15.7% from 70 to 79.

**Table 3:** The research participants’ usage patterns and preferences for personal listening devices.

Patterns and preferences for personal listening devices		*N*	%
What type of headphones do you prefer to use?	Car speakers	73	17.1%
	Earphones	234	54.7%
	Headphones	83	19.4%
	Large external speakers	38	8.9%
How many times do you use these headphones during the week?	1–5 times	185	43.2%
	6–9 times	106	24.8%
	More than 10 times	92	21.5%
	Never	45	10.5%
How much time do you spend listening with this headphone during the day?	An hour or less	184	43.0%
	From 3 h to 5 h	70	16.4%
	More than 5 h	41	9.6%
	1–2 h	133	31.1%
Are people near and around you disturbed by the sounds coming from these headphones?	Never	247	57.7%
	Sometimes	136	31.8%
	Usually	32	7.5%
	Always	13	3.0%
What volume level do you usually prefer?	0–49	119	27.8%
	50–59	91	21.3%
	60–69	76	17.8%
	70–79	67	15.7%
	80–89	40	9.3%
	90–100	35	8.2%

**Figure 1:**
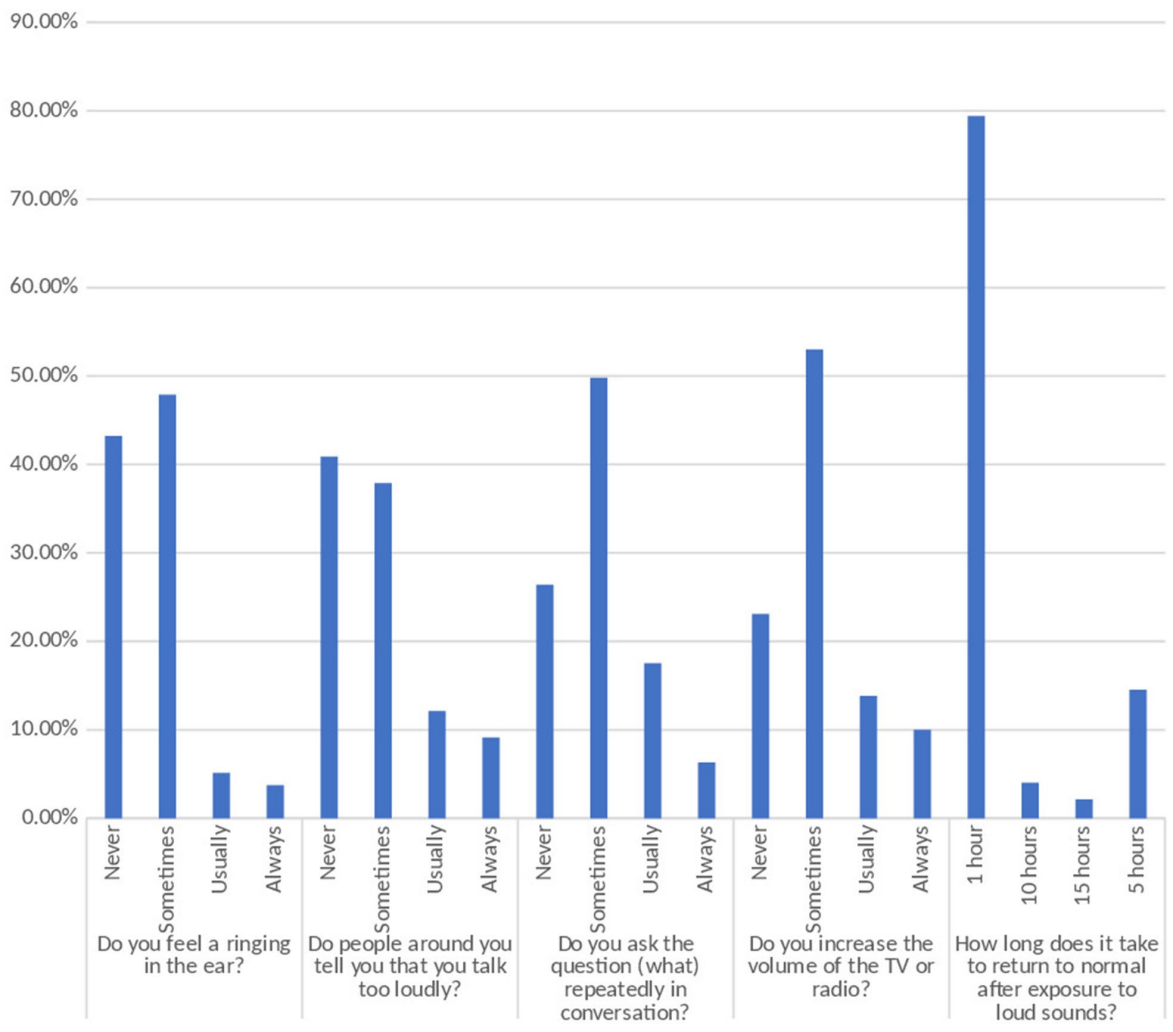
Hearing-related symptoms and behaviors as described by the individual.

Figure 1 displays the participants’ self-reported hearing-related symptoms and behaviors. Nearly half of the subjects (47.9%) reported a ringing sensation in their ears, 5.1% generally experienced it, and 3.7% always experienced it. Significantly more participants (37.9%) said that they occasionally heard complaints from individuals around them that they spoke too loudly, compared to 12.1% who said they frequently heard such remarks and 9.1% who claimed they always did. Approximately 49.8% of participants asked the question “what” many times during a talk, compared to 17.5% who did so often and 6.3% who did it continually. Of the participants, 53.0% occasionally increased the volume of TV or radio, 13.8% did so on a regular basis, and 10.0% did so continually. When asked how long it took to regain normal hearing after being exposed to loud noises, most participants (79.4%) indicated 1 h, 14.5% who said 5 h, 4.0% who said 10 h, and 2.1% claimed 15 h, for further details refer to Supplementary Table S1.

Table 4 shows the participants’ understanding of and attitudes toward prevention. Of the participants, 81.1% believed that loud noises were harmful to their hearing, whereas 10.7% expressed doubts. Similarly, 76.4% of individuals reported that their ability to hear was hampered by working or living in a loud environment, while 12.1% expressed doubts. 13.8% of people expressed uncertainty, whereas the majority (77.6%) believed that extended exposure to loud noises may worsen HL. Around 27 % of subjects indicated confusion, while the majority (56.1%) reported hearing certain words faintly or having trouble interpreting them during discussions as the first sign of HL. Around 37 % of individuals voiced suspicion, whereas less than half (43.9%) believed that ringing in the ears indicated HL. Sixty-one percent of the participants believed that a regular increase in the volume of radio or television indicated deterioration in hearing capacity, whereas others were not convinced.

**Table 4:** Knowledge and attitudes on noise-induced hearing loss.

Knowledge and attitudes		*N*	%
In your opinion, do high sound levels affect hearing?	No	35	8.2%
	Yes	347	81.1%
	I do not know	46	10.7%
In your opinion, does living or working in a noisy environment affect hearing?	No	49	11.4%
	Yes	327	76.4%
	I do not know	52	12.1%
In your opinion, can hearing loss get worse when listening to loud sounds for a long time?	No	37	8.6%
	Yes	332	77.6%
	I do not know	59	13.8%
In your opinion, is not understanding some words or hearing them in a low or faint way while speaking with others considered an early sign of hearing loss?	No	74	17.3%
	Yes	240	56.1%
	I do not know	114	26.6%
In your opinion, is the feeling of ringing in the ear a sign of hearing loss?	No	81	18.9%
	Yes	188	43.9%
	I do not know	159	37.1%
In your opinion, does a continuous increase in the volume of the television or radio indicate the presence of hearing loss?	No	83	19.4%
	Yes	261	61.0%
	I do not know	84	19.6%
In your opinion, can hearing loss problems caused by noise be prevented?	No	35	8.2%
	Yes	298	69.6%
	I do not know	95	22.2%
Do you currently have enough information about the risk that exposure to loud sounds poses to your hearing?	No	184	43.0%
	Yes	244	57.0%
In your opinion, what is the minimum possible period of exposure to loud sound from any source that could lead to hearing loss?	30 min	122	28.5%
	1 h	60	14.0%
	One and a half hours	50	11.7%
	2 h or more	81	18.9%
	I do not know	115	26.9%
In your opinion, what is the lowest sound level could lead to hearing loss?	20–40	64	15.0%
	41–60	46	10.7%
	61–80	72	16.8%
	81–90	71	16.6%
	91–100	57	13.3%
	I do not know	118	27.6%
In your opinion, what is the best source that can be used to learn more about the problem of hearing loss resulting from exposure to noise?	Educational campaigns	107	25.0%
	Hospitals	133	31.1%
	Schools and the work environment	58	13.6%
	Shopping Center	21	4.9%
	Social media sites	88	20.6%
	Television	21	4.9%

Of all participants, 69.6% believed that noise-induced HL could be avoided, while 22.2% were unclear. Most participants (57.0%) demonstrated adequate knowledge of the possible hearing impairment induced by loud sounds. Among the participants, 28.5% believed that exposure to loud noise for at least 30 min may result in HL, whereas 26.9% were unclear. Participants had a variety of ideas about the minimum sound level that might induce HL, with 27.6% expressing doubt. Hospitals (31.1%), educational campaigns (25.0%), social networking sites (20.6%), workplaces and schools (13.6%), and television and retail outlets (4.9%) were the most effective venues for learning NIHL.

Table 5 displays the viewpoints and choices of the participants regarding the use of personal listening devices to mitigate NIHL. During the bulk of their listening time, 72.9% of participants opted to decrease the volume of their devices. Approximately three-quarters (75.9%) of the participants reported receiving guidance from the manufacturer of their personal headphones to mitigate the risk of excessive noise exposure by utilizing the safe volume feature. Of all the participants, 11.0% expressed uncertainty. Off the participant, 86.7% suggested implementing safe volume warnings on audio equipment. Upon being presented with information indicating that listening to loud noises may potentially damage their hearing, a majority of the participants (56.3%) expressed a consistent willingness to modify their habit of increasing the volume of their headphones. Additionally, 18.2% indicated a general inclination to do so, whereas 18.7% expressed occasional willingness. Similarly, 54.0% of individuals consistently favored using software to establish acceptable sound levels for themselves and their families, while 20.1% preferred using it frequently, and 17.1% chose to use it sometimes.

**Table 5:** Attitudes and preferences for preventing noise-induced hearing loss.

Attitudes and preferences		*N*	%
Do you prefer to reduce the volume on your device most of the listening time?	No	116	27.1%
	Yes	312	72.9%
Does the manufacturer of your personal headphones advise you to set a safe volume feature to limit exposure to high, harmful sound levels?	No	56	13.1%
	Yes	325	75.9%
	I do not know	47	11.0%
Do you recommend placing safe volume warning indicators on audio devices?	No	57	13.3%
	Yes	371	86.7%
Are you ready to change your habit of turning up your headphones if you hear or see evidence that listening to loud sounds harms your hearing?	Never	29	6.8%
	Sometimes	80	18.7%
	Usually	78	18.2%
	Always	241	56.3%
Would you prefer to use software to determine safe sound levels for you and your family?	Never	38	8.9%
	Sometimes	73	17.1%
	Usually	86	20.1%
	Always	231	54.0%

Table 6 displays the distribution of participants’ demographics and health-related characteristics of the participants and their HL. No significant differences were found between sex (*p* = 0.677), age (*p* = 0.254), marital status (*p* = 0.225), place of residence (*p* = 0.138), degree of education (*p* = 0.082), employment (*p* = 0.295), and HL. However, a statistically significant difference was observed between nationality and hearing impairment (*p* = 0.040). This study revealed a significant difference between smoking status and hearing impairment (*p* = 0.007). The percentage of participants experiencing HL was higher among individuals who quit smoking (16.4% vs. 6.8%) and continued to smoke (26.2% vs. 18%). This study revealed a substantial difference between comorbidities and HL (*p* < 0.001). The prevalence of diabetes (14.8% vs. 3.8%) and hypertension (14.8% vs. 4.1%) significantly differed between participants who have HL and those who do not. A higher percentage of individuals with a familial background of noise-induced HL (59.0% vs. 42.5%) experienced hearing impairments. A significant difference was found between HL and familial predisposition to NIHL (*p* = 0.016).

**Table 6:** Hearing loss status stratified by demographic and health-related variables.

Factors		Hearing loss				*p*-value
		No		Yes		
		*N*	%	*N*	%	
Gender	Male	194	52.9%	34	55.7%	0.677
	Female	173	47.1%	27	44.3%	
Age	18–25	171	46.6%	24	39.3%	0.254
	26–35	97	26.4%	17	27.9%	
	36–50	80	21.8%	13	21.3%	
	Over 50	19	5.2%	7	11.5%	
Marital status	Single	198		38	62.3%	0.225
	Married	169	46.0%	23	37.7%	
Residence	City	179	48.8%	36	59.0%	0.138
	Village	188	51.2%	25	41.0%	
Education level	Middle or below	20	5.4%	2	3.3%	0.082
	Secondary	66	18.0%	11	18.0%	
	University	261	71.1%	39	63.9%	
	Postgraduate	20	5.4%	9	14.8%	
Employment	Health practitioner	140	38.1%	19	31.1%	0.295
	Outside the health field	227	61.9%	42	68.9%	
Smoking	No	276	75.2%	35	57.4%	0.007
	Yes	66	18.0%	16	26.2%	
	Quit smoking	25	6.8%	10	16.4%	
Comorbidities	Diabetes	14	3.8%	9	14.8%	<0.001
	Hypertension	15	4.1%	9	14.8%	
	Heart disease	7	1.9%	1	1.6%	
	None	331	90.2%	42	68.9%	
Family history of NIHL	No	211	57.5%	25	41.0%	0.016
	Yes	156	42.5%	36	59.0%	

Table 7 illustrates the difference between noise exposure, headphone usage, and other pertinent behaviors among individuals participating in the study and the occurrence of HL. A significant difference (*p* = 0.002) was observed between occupational noise exposure and hearing impairment, with a higher proportion of individuals (54.1% versus 33.8%) being susceptible to HL. There was no statistically significant difference between the type of headphones used (*p* = 0.844), frequency of headphone uses throughout the week (*p* = 0.779), duration of headphone uses during the day (*p* = 0.537), and HL. However, a statistically significant difference (*p* = 0.026) was observed between hearing impairment and the use of headphones that annoyed others in the vicinity. Individuals who wore headphones daily (9.8%) or seldom (45.9%) were more likely to disturb individuals with hearing impairments.

**Table 7:** Hearing loss and noise exposure, headphone use, and associated behaviors.

Factor		Hearing loss				*p*-value
		No		Yes		
		*N*	%	*N*	%	
Are you exposed to noise at your work?	No	243	66.2%	28	45.9%	0.002
	Yes	124	33.8%	33	54.1%	
What type of headphones do you prefer to use?	Car speakers	64	17.4%	9	14.8%	0.844
	Earphones	202	55.0%	32	52.5%	
	Headphones	69	18.8%	14	23.0%	
	Large external speakers	32	8.7%	6	9.8%	
How many times do you use these headphones during the week?	1–5 times	160	43.6%	25	41.0%	0.779
	6–9 times	88	24.0%	18	29.5%	
	>10 times	79	21.5%	13	21.3%	
	Never	40	10.9%	5	8.2%	
How much time do you spend listening with this headphone during the day?	<1 h	163	44.4%	21	34.4%	0.537
	1-2 h	111	30.2%	22	36.1%	
	3-5 h	59	16.1%	11	18.0%	
	>5 h	34	9.3%	7	11.5%	
Are people near and around you disturbed by the sounds coming from these headphones?	Never	222	60.5%	25	41.0%	0.026
	Sometimes	108	29.4%	28	45.9%	
	Usually	26	7.1%	6	9.8%	
	Always	11	3.0%	2	3.3%	
What volume level do you usually prefer?	0–49	106	28.9%	13	21.3%	0.197
	50–59	83	22.6%	8	13.1%	
	60–69	62	16.9%	14	23.0%	
	70–79	55	15.0%	12	19.7%	
	80–89	31	8.4%	9	14.8%	
	90–100	30	8.2%	5	8.2%	
Do you increase the volume of the TV or radio?	Never	89	24.3%	10	16.4%	0.064
	Sometimes	198	54.0%	29	47.5%	
	Usually	48	13.1%	11	18.0%	
	Always	32	8.7%	11	18.0%	

### The association between demographics, headphone use pattern and hearing loss score

The mean HL score was 8.9 (SD: 2.8) out of 20; which reflects low level of HL among the study participants. Males were more likely to have higher level of HL compared to females (odds ratio: 1.61 (95% confidence interval (CI): 1.10–2.36); *p* = 0.015). Participants who prefer high volume level (above 70 dB) were more than 2-folds (odds ratio: 2.55 (95%CI: 1.37–4.75)) likely to have higher level of HL compared to others (*p* < 0.01), Table 8.

**Table 8:** Predictors of hearing loss.

Variable		Odds ratio of having higher level of hearing loss	*p*-value
Gender	Male (Reference category)	1.00	
	Female	0.62 (0.42–0.91)	0.015*
Age	18–25 (Reference category)	1.00	
	26–35	1.50 (0.93–2.40)	0.097
	36–50	0.75 (0.46–1.27)	0.264
	Over 50	0.31 (0.12–0.77)	0.012*
Nationality	Saudi (Reference category)	1.00	
	Non-Saudi	1.30 (0.36–4.67)	0.689
Marital status	Single (Reference category)	1.00	
	Married	0.71 (0.48–1.03)	0.074
Residence	City (Reference category)	1.00	
	Village	1.14 (0.78–1.67)	0.493
Education level	Middle school or below (Reference category)	1.00	
	Secondary	0.20 (0.06–0.73)	0.015*
	University	0.15 (0.05–0.53)	0.003**
	Postgraduate	0.35 (0.08–1.50)	0.157
Employment	Health practitioner (Reference category)	1.00	
	Outside the healthcare field	1.10 (0.75–1.63)	0.624
What type of headphones do you prefer to use?	Car speakers (Reference category)	1.00	
	Earphones	0.58 (0.34–0.99)	0.046*
	Headphones	0.85 (0.44–1.61)	0.612
	Large external speakers	0.59 (0.27–1.30)	0.188
How many times do you use these headphones during the week?	1–5 times (Reference category)	1.00	
	6–9 times	1.28 (0.79–2.07)	0.318
	>10 times	1.03 (0.63–1.71)	0.897
	Never	0.87 (0.45–1.67)	0.670
How much time do you spend listening with this headphone during the day?	<1 h (Reference category)	1.00	
	1-2 h	1.44 (0.92–2.25)	0.115
	3-5 h	1.31 (0.76–2.29)	0.333
	>5 h	1.48 (0.74–2.93)	0.267
What volume level do you usually prefer?	0–49 (Reference category)	1.00	
	50–59	1.42 (0.82–2.47)	0.206
	60–69	1.74 (0.97–3.11)	0.062
	70–79	2.55 (1.37–4.75)	0.003*
	80–89	1.63 (0.79–3.35)	0.184
	90–100	2.56 (1.16–5.61)	0.019*

*p > 0.05; **p > 0.01.

## Discussion

The current study sought to determine the awareness of NIHL and its association with PLD use among residents in the Jazan region of Saudi Arabia. In this study, a significant proportion of participants (81.1%) had knowledge that loud noises can lead to hearing issues. Nevertheless, there is a substantial disparity between knowledge and implementation, as some individuals persist in engaging in behaviors that have the potential to harm their auditory faculties. This demonstrates that awareness level does not necessarily reflect that the individuals implementing preventative measures. This study examined susceptibility to hearing impairment and several demographic and health-related variables. In our study, the proportion of smokers who have HL was higher compared to non-smokers (*p*-value = 0.007), indicating that individuals who smoke or had smoked in the past were at greater risk of NIHL. Previous studies have found that smoking is significantly associated with HL (21–24). Moreover, a study by Kumar et al. found that smokers were more susceptible to sensorineural type of HL, with the mild type (26–40 dB) of HL being the most common one (24). Nicotine-induced vasospasms, thrombotic occlusions, and atherosclerotic blood vessel narrowing, all are factors that contribute to the reduction of blood supply as a result of smoking, which ultimately lead to HL (25).

In our study HL was more common across patients with comorbidities such as hypertension and diabetes mellitus (*p*-value <0.001). Previous literature confirmed that comorbidities such as cardiovascular diseases, diabetes mellitus, hypertension, and dyslipidemia are risk factors for HL through oxidative stress and chronic inflammation (26–30). Co-existed conditions like diabetes mellitus and hypertension could affect the auditory system through multiple mechanisms including diminished blood flow, nerve damage, and microvascular damage (27, 29).

According to our research, a sizable majority of participants are aware of the possible dangers associated with loudly using personal listening devices. This high level of awareness is demonstrated by the data, which indicates that the majority of respondents prefer to listen at lower volume levels and that many of them choose to limit their exposure to noise levels that may be harmful. This is in contrast to past research conducted in areas where there was a notable lack of knowledge regarding NIHL. Similar studies conducted in Saudi Arabia have shown varying levels of awareness and behaviors concerning NIHL. For instance, a study in Makkah found that 22% of subjects had mild-to-severe hearing impairment, yet most preferred lower volume levels as a preventive measure (12). Another study in Hail revealed significant hearing impairments due to low awareness levels (13). These findings align with our study, where despite high awareness, the implementation of preventive measures remains inadequate.

In our study, almost half of the participants (47.9%) reported experiencing a ringing sensation in their ears. This finding aligns with a study carried out in Makkah region, Saudi Arabia, where (40%) of participants reported tinnitus or ringing in the ear (12). In contrast, a study conducted in Jordan revealed that (21%) of the participants reported such sensations (18). This discrepancy suggests that tinnitus may serve as an indicator of both NIHL and other hearing health problems. Given that the majority of the subjects in our study are young aged 18–25 years, compared to the Makkah study (15) which the majority over 50 years old, tinnitus could point toward different hearing problems in various age groups. Consequently, it is critical to evaluate the ears in individuals who complain of tinnitus, regardless of their age. Furthermore, the majority of participants (54.7%) indicated a preference for using earphones. Similar trends have been noted in Malaysia and Makkah region, Saudi Arabia, where (51%) and (45.3%) of participants, respectively, reported using earphones. Approximately (43.0%) of the current study’s participants listened to a noise source for less than 1 h per session (12). In contrast to our study, two studies in Malaysia reported longer mean listening times [1.2 ± 1.5 h (31) and 1.5–3.2 h (12)]. Cultural and religious influences likely play a role in the comparatively shorter periods of personal audio device (PAD) usage observed in the Saudi community. Nevertheless, this study reported that (14.3%) of participants had hearing impairment. Similarly at a high level close to the study conducted in Makkah region, Saudi Arabia, which found that (24.8%) of the participants reported a hearing impairment (12). These findings in Saudi Arabia studies were much higher than those findings reported in American study (10%) and Malaysian study (7.3%) (31, 32).

The mean HL score among our study participants was 8.9 (SD: 2.8) out of 20; which reflects low level of HL among the study participants. In our study, males were 1.6 folds more likely to have higher level of HL compared to females (*p* = 0.015). The 2019 National Health Interview Survey data looked at adult hearing impairments in the US. They observed that among individuals 45 and older, males were more likely than females to report having some degree of HL or not being able to hear at all (33). Males were more likely than females in all age groups to use a hearing aid in 2019, with 7.1% of people 45 and older reporting such use (33). One of the main cause is that males are more likely than females to be exposed to loud noises. This is confirming the findings of our study where HL was more common across those exposed to workplace noise (*p*-value = 0.002).

In industries that were traditionally dominated by males, such as construction, manufacturing, and the military, there is greater gender disparity (33, 34). Individuals in any of these occupations may be subjected to loud noises such as heavy machinery, power tools, and even shooting. In the event that one is not properly protected, exposure to loud noises over an extended period of time can lead to HL in later life (33, 34). On the other hand, other literature has demonstrated that males are biologically more susceptible to NIHL than females, and this is not solely due to certain occupations (35). Previous animal model studies demonstrated that female mice are significantly protected from NIHL in comparison to males and that males are more noise vulnerable (36, 37). Besides, previous study established that there is a considerable male–female difference in noise-induced permanent threshold shift, even though both groups were exposed to the same Equivalent Continuous Sound Level (Leq) (38). Moreover, a study by Wang et al. found that males are at higher risk of high-frequency hearing loss compared to females (39). Another study examined factors associated with hearing impairment (HI) in adolescent youths between 1966 and 2010 (23). This study concluded that HI increased twofold for males and cigarette smoking (23). Another important factor that might have contributed to low level of awareness concerning the use of PLDs and NIHL is the culture. In the Middle East region, loud social gathering and celebrations are common without adequate preventive measure due to limited emphasis on public health education directed toward hearing health. A previous study reported that the noise levels in most amusement arcades have exceeded the exposure limits recommended by the Environment Protection Agency (40).

Participants who prefer high volume level (above 70 dB) were more than 2-folds likely to have higher level of HL compared to others (*p* < 0.01). In addition, approximately 81.1% of the participants expressed a belief that high volume levels could have a negative impact on their hearing. This finding is consistent with two studies conducted in Saudi Arabia, one in the Makkah region where 89% of respondents believed that high volume levels could harm their hearing, and the other in the Hail region where 69.6% shared this belief. These results suggest a significant level of awareness regarding the effects of sound levels on hearing in Saudi Arabia (12, 13). Even with prolonged exposure, sounds at or below 70 A-weighted dBA are unlikely to result in HL (41). Hearing loss, however, can result with prolonged or recurrent exposure to noise levels of 85 dBA or above. The duration of time it takes for NIHL to occur increases with sound intensity (41).

Public health initiative should launch educational campaigns to enhance public awareness concerning the use of PLDs and NIHL and reduce the incidence of NIHL. This initiative should target high risk population such as males and those who use PLDs more frequently such as school and university students. Besides, social media platforms should promote the safe use of PLDs. From clinical perspective, healthcare professionals should incorporate routine hearing health checks more in their practices. This is an important step toward early detection and prevention.

### Strengths and limitations

This study utilized a comprehensive questionnaire that covered various relevant aspects, from personal data to medical history and PLD usage patterns. However, our study’s limitations include the use of convenience sampling, a non-random method, which may restrict the generalizability of our study to the broader Jazan population. The lack of direct hearing assessments, such as otologic and audiometric testing, limits our ability to objectively evaluate the prevalence of NIHL among the participants. Future studies are recommended to examine the association between the use of PLD and NIHL incorporating clinical assessment for hearing loss.

Also, the cross-sectional nature of our study provides a snapshot in time but does not allow for the examination of causal relationships or changes over time. Besides, this study did not collect data on ear-related conditions history (such as genetic issues, cholesteatoma, or recurrent ear infections). Prior ear-related conditions are important aspect that should be taken into consideration for interpreting susceptibility to hearing loss in future research. Future studies may benefit from incorporating such data to provide a more comprehensive understanding of the multifactorial contributors to hearing outcomes. These limitations should be taken into consideration when interpreting our study’s findings and designing future research in this area.

## Conclusion

This study reveals a moderate level of awareness of NIHL and the associated risks of PLDs among residents of the Jazan region, Saudi Arabia. Despite that, translating knowledge into preventive action remains a challenge, particularly among younger demographics who are more frequent users of PLDs. The findings of our study underscore the need for continued and targeted public health interventions that go beyond raising awareness to fostering lasting behavioral change and reduce the incidence of NIHL. Future research should explore the long-term effectiveness of these interventions and how technology can be utilized to strengthen hearing conservation strategies. By addressing these gaps, there is potential to significantly reduce the incidence of NIHL in the region and set a precedent for similar initiatives across Saudi Arabia.

## Data Availability

The original contributions presented in the study are included in the article/Supplementary material, further inquiries can be directed to the corresponding author.
