# Supplementary material for: Awareness of and the relationship between noise-induced hearing loss and the use of personal listening devices in Jazan region, Saudi Arabia
Source: Front Public Health. 2025 Feb 21;13:1505442. doi: 10.3389/fpubh.2025.1505442 (PMC11885267; doi:10.3389/fpubh.2025.1505442)
Supplement: Supplementary file 1 [file Table_1.docx]

Supplementary material:

Table S1: Distribution of signs and symptoms related to noise induced hearing loss

|  | Never | Sometimes | Usually | Always |
| --- | --- | --- | --- | --- |
| Ringing in the ears | 185 (43.2%) | 205 (47.9%) | 22 (5.1%) | 16 (3.7%) |
| People said I talk loud | 175 (40.9%) | 162 (37.9%) | 52 (12.1%) | 39 (9.1%) |
| I tend to ask “What?” repeatedly in a conversation | 113 (26.4%) | 213 (49.8%) | 75 (17.5%) | 27 (6.3%) |
| Increasing the volume of the TV or radio is something I do | 99 (23.1%) | 227 (53.0%) | 59 (13.8%) | 43 (10.0%) |
|  | 1 h | 5 hrs | 10 hrs | 15 hrs |
| Time I need to adapt with surrounding environmental sound when exposed to  loudness (h) | 340 (79.4%) | 62 (14.5%) | 17 (4.0%) | 9 (2.1%) |
